# Evaluating the clinical utility of semi-quantitative luciferase immunosorbent assay using *Treponema pallidum* antigens in syphilis diagnosis and treatment monitoring

**DOI:** 10.1080/22221751.2024.2348525

**Published:** 2024-04-25

**Authors:** Wujian Ke, Cailing Ao, Ran Wei, Xiaozhen Zhu, Jingwei Shui, Jianhui Zhao, Xiaohui Zhang, Liuyuan Wang, Liping Huang, Xinying Leng, Rui Zhu, Jiaxin Wu, Lixia Huang, Nanxuan Huang, Haiying Wang, Wenjia Weng, Ligang Yang, Shixing Tang

**Affiliations:** a Department of Sexually Transmitted Diseases, Dermatology Hospital of Southern Medical University, Guangzhou, Guangdong Province, People’s Republic of China; b Department of Epidemiology, School of Public Health, Southern Medical University, Guangzhou, Guangdong Province, People’s Republic of China; c Guangzhou Baiyun District Center for Disease Control and Prevention, Guangzhou, Guangdong Province, People’s Republic of China; d Department of Dermatology, Tianjin Children Hospital, Tianjin, People’s Republic of China; e Center of Clinical Laboratory, Zhongshan Hospital, Medical College of Xiamen University, Xiamen, Fujian Province, People’s Republic of China; f Institute of Infectious Disease, Medical College of Xiamen University, Xiamen, Fujian Province, People’s Republic of China; g Department of Dermatology, Beijing Youan Hospital, Capital Medical University, Beijing, People’s Republic of China

**Keywords:** *Treponema pallidum*, treponemal antibodies, TP0171 (TP15), TP0435 (TP17), TP0574 (TP47), luciferase immunosorbent assay (LISA), syphilis diagnosis, treatment monitoring

## Abstract

To assess the clinical applicability of a semi-quantitative luciferase immunosorbent assay (LISA) for detecting antibodies against *Treponema pallidum* antigens TP0171 (TP15), TP0435 (TP17), and TP0574 (TP47) in diagnosing and monitoring syphilis. LISA for detection of anti-TP15, TP17, and TP47 antibodies were developed and evaluated for syphilis diagnosis using 261 serum samples (161 syphilis, 100 non-syphilis). Ninety serial serum samples from 6 syphilis rabbit models (3 treated, 3 untreated) and 110 paired serum samples from 55 syphilis patients were used to assess treatment effects by utilizing TRUST as a reference. Compared to TPPA, LISA-TP15, LISA-TP17, and LISA-TP47 showed a sensitivity of 91.9%, 96.9%, and 98.8%, specificity of 99%, 99%, and 98%, and AUC of 0.971, 0.992, and 0.995, respectively, in diagnosing syphilis. Strong correlations (*r_s_* = 0.89–0.93) with TPPA were observed. In serial serum samples from rabbit models, significant differences in the relative light unit (RLU) were observed between the treatment and control group for LISA-TP17 (days 31–51) and LISA-TP47 (day 41). In paired serum samples from syphilis patients, TRUST titres and the RLU of LISA-TP15, LISA-TP17, and LISA-TP47 decreased post-treatment (*P* < .001). When TRUST titres decreased by 0, 2, 4, or ≥8-folds, the RLU decreased by 17.53%, 31.34%, 48.62%, and 72.79% for LISA-TP15; 8.84%, 17.00%, 28.37%, and 50.57% for LISA-TP17; 22.25%, 29.79%, 51.75%, and 70.28% for LISA-TP47, respectively. Semi-quantitative LISA performs well for syphilis diagnosis while LISA-TP17 is more effective for monitoring syphilis treatment in rabbit models and clinical patients.

## Introduction

Syphilis is a chronic, systemic sexually transmitted disease (STD) caused by the *Treponema pallidum* (*T. pallidum*). It is one of the most prevalent STDs in the world. In the United State, the cases of syphilis increase by 32% during 2020–2021 [1]. In China, from 2007 to 2017, the morbidity of syphilis presented a significant rise from 15.9/100,000 to 34.5/100,000 [2]. Therefore, early and accurate diagnosis of syphilis is crucial in reducing its incidence.

Currently, the laboratory diagnosis of syphilis mainly relies on the detection of two types of antibodies through serological tests: the non-treponemal tests (NTTs) and the treponemal tests (TTs). The NTTs include the rapid plasma reagin (RPR), Venereal Disease Research Laboratory (VDRL), and toluidine red unheated serum test (TRUST), and are usually used to reflect disease activity and monitor response to treatment [3]. However, NTTs can lead to false positivity in conditions such as autoimmune diseases, malignancy, and intravenous drug use [4]. There are also other drawbacks for NTTs, including (1) the inability to evaluate the effectiveness of the treatment when the baseline NTT titres is low (less than 1:4) [3]; (2) the “serofast” result, in which the antibody titres may not decrease after treatment, that could result in over-treatment and unnecessary examination [5]; (3) unusual increase of NTT titres in some individuals post-treatment [6]; (4) unusual decrease of NTT titres in some individuals without treatment [7]. These disadvantages underscore the importance of finding new specific indicators and assays for monitoring the efficacy of syphilis treatment.

The TTs include the fluorescent treponemal antibody absorption (FTA-Abs), *T. pallidum* particle agglutination (TPPA), enzyme immunoassay (EIA), and chemiluminescence immunoassay (CLIA). These tests are primarily used for the diagnosis of syphilis, but cannot be used to evaluate the effectiveness of treatment [8]. However, recent studies have explored the dynamics of treponemal antibody levels as a method of assessing the response to treatment. For example, the reaction intensities of TP17 and TP47 antibodies tested by Western Blot could potentially be used to monitor the therapeutic effect of syphilis [9], though further research is needed to examine the correlation between the change in treponemal antibody levels and the treatment efficacy by conducting quantitative TTs on paired follow-up serum samples from syphilis patients.

We have previously developed a highly sensitive and efficient luciferase immunosorbent assay (LISA) to detect specific IgG against HIV-1 [10]. This assay is 10^4^ times more sensitive than enzyme-linked immunosorbent assay (ELISA) and can be used to monitor the changes of anti-HIV antibody titres following combined antiretroviral therapy (cART) [11]. The LISA system has also been used to detect antibodies against Zika virus, *Chlamydia trachomatis*, SARS-CoV-2, norovirus, and Chikungunya virus [12–16]. In this study, we explored LISA to detect specific IgG against three highly immunogenic antigens of *T. pallidum*, i.e. TP15, TP17, and TP47, and evaluated its syphilis diagnostic performance using TPPA as a reference since TPPA is a treponema-specific test for the diagnosis of syphilis and is more sensitive than FTA-Abs in primary syphilis patients [17]. TPPA can quantitatively measure the level of antibody and be reported as titres, making it suitable for comparison with the semi-quantitative LISA. In addition, TPPA is recommended for adjudicating discordant results between the treponemal immunoassay and NTTs [18].

Compared with the commercial TPPA assay, LISA has several advantages, including semi-quantitative capability, less laborious when combined with an automated system for incubation and washing [19,20]. To further evaluate the effectiveness of LISA in monitoring syphilis treatment efficacy, the levels of TP15, TP17, and TP47 antibodies were measured using LISA in serial specimens from syphilis rabbits or paired follow-up serum samples from syphilis patients pre- and post-treatment. Our goal was to determine whether LISA could be used in the diagnosis of syphilis and the evaluation of treatment efficacy.

## Methods

### Samples

#### Samples of syphilis patients and blood donors for syphilis diagnosis

To evaluate the diagnostic performance of LISA for syphilis, we collected serum samples from 201 participants who underwent TPPA and TRUST testing at the STD clinic in the Dermatology Hospital of Southern Medical University from December 2019 to December 2021 and 60 serum samples from healthy blood donors (Supplemental Table 1). These samples consisted of 161 TPPA-positive and 40 TPPA-negative samples from STD clinic, as well as 60 serum samples from TPPA-negative blood donors. The TPPA assay (Fujirebio, Japan) and TRUST (Rongsheng, China) were performed according to the manufacturer’s instructions.

#### Samples collected from T. pallidum-infected New Zealand white rabbits

To evaluate the change in the levels of treponemal antibodies against TP15, TP17 and TP47 detected by LISA with comparison to TRUST, we used 90 serial serum samples from 6 New Zealand white rabbits infected with *T. pallidum* (divided into Benzathine Penicillin G (BPG)-treated and untreated groups), detailed in Supplemental Figure 1.

#### Samples from patients with different stages of syphilis for assessing treatment response

To determine the change in the antibody responses of LISA-TP15, LISA-TP17 and LISA-TP47 to treatment, paired serum samples were collected from 55 patients with different stages of syphilis before and after antibiotic therapy. Participants were recruited from the Dermatology Hospital of Southern Medical University between December 2019 and December 2021. Data of demographics, laboratory tests by using commercial kits, and treatment information were extracted from electronic medical records (detailed in Supplemental Table 4).

### Development of LISA

**Figure 1.**
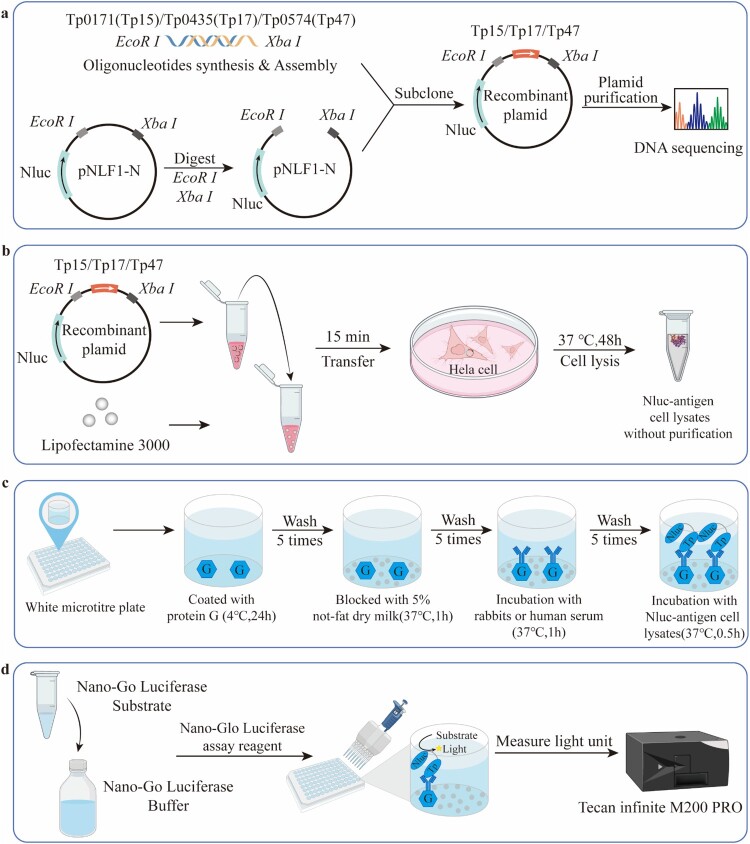
Schematic of the steps involved in LISA. (a) The gene sequences of Tp0171 (Tp15), Tp0435 (Tp17), and Tp0574 (Tp47) were obtained from GenBank (Genbank ID: AE000520.1) and synthesized with *EcoRI* and *XbaI* cleavage sites. The sequence of Tp15, Tp17, and Tp47 were cloned into a pNLF1-N vector containing the NanoLuc (Nluc) luciferase gene. (b) The resulting recombinant plasmid was expressed in Hela cells to produce Nluc-antigen fusion proteins. (c) A white microtitre plate coated with protein G was used to bind antibodies from either rabbits or human serum. The Nluc-antigen fusion proteins were then employed to specifically bind with IgG antibodies against the TP15, TP17, or TP47 antigens. (d) The light units were measured using the Nano-Glo Luciferase assay reagent in a luminometer to reflect the level of antibodies.

The LISA methodology has previously been described [10–16]. We optimized the expression of *T. pallidum* fusion proteins of Nanoluc (Nluc) – TP15, TP17, and TP47 in Hela cells. The gene sequences of Tp0171 (Tp15), Tp0435 (Tp17), and Tp0574 (Tp47) were obtained from GenBank (Genbank ID: AE000520.1) and synthesized with *EcoRI* and *XbaI* cleavage sites (Sangon Biotech Co., Ltd, China). The gene fragments were cloned into the *EcoRI*-*XbaI*-cut pNLF1-N vector with the Nluc luciferase gene. The integrity of the Nluc-antigen fusion constructs was confirmed through DNA sequencing (Sangon Biotech Co., Ltd, China). HeLa cells were plated at a density of 10^6^ cells per dish (10-cm diameter) and transfected with 5 μg plasmid DNA by using Lipofectamine 3000 (Invitrogen, USA). After 48 h, the cells were lysed and the lysates, containing Nluc-TP15/Nluc-TP17/Nluc-TP47 fusion proteins, were harvested and stored at −80°C for future use without further purification. To determine the activity and expression level of fusion proteins, luciferase activity of crude cell extracts was measured after adding an equal volume of Nano-Glo Luciferase assay reagent (Promega, USA) in a luminometer (Tecan infinite M200 PRO, Switzerland). To detect TP15, TP17, and TP47 antibodies, white microtitre plates were coated with 5 μg/mL of protein G (Genscript Co., China) in 0.01 M PBS and incubated overnight at 4°C. The plates were then washed 5 times with 0.01 M PBS containing 0.05% Tween 20 (PBS-T) and blocked with 5% non-fat dry milk (NFDM) for 1 h at 37°C and then washed 5 times with PBS-T. 50 μL rabbit or human serum samples (1:100 dilution in 2% NFDM) were added to the plates and incubated for 1 h at 37°C. After washing with PBS-T 5 times, 50 μL Nluc-antigens crude cell extract was then added to each well for 0.5 h at 37°C. After washing with PBS-T 5 times, 50 μL Nluc-antigens crude cell extract was then added to each well for 0.5 h at 37°C. After washing with PBS-T 5 times, 50 μL Nano-Glo Luciferase assay reagent was then added to each well. The light unit (LU) was determined within 2 h using a luminometer. The average LU of parallel duplicate tests for each sample was recorded as crude LU, divided by the average LU of negative controls, and presented as relative light unit (RLU) (Figure 1).

### Data analysis

The receiver operating characteristic (ROC) analysis and the area under curve (AUC) were used to evaluate the performance of LISA. The optimal cut-off value was determined using the maximum Youden’s index. The confidence intervals (CIs) of 95% were calculated for sensitivity, specificity, positive likelihood ratio (+LR), and negative likelihood ratio (−LR) in the ROC analysis. Additionally, a Spearman’s rank correlation was adapted to analyse the correlation between the results of LISA and TPPA tests.

Antibody levels were expressed as mean ± standard deviation (SD) while the antibody titres differences between the two rabbit groups were tested using a Student’s *t*-test. The median percent change in antibody levels against TP15, TP17, and TP47 detected by LISA in the paired follow-up syphilis patients’ samples was calculated using the Hodges–Lehmann estimate and stratified by the changes of TRUST titres. A Spearman’s rank correlation analysis was conducted to examine the relationship between changes in serum antibody levels against TP15, TP17, and TP47 as tested by LISA and changes in TRUST titres. Statistical analysis was performed using SPSS 25.0 and MedCalc software, and a *P*-value less than .05 was considered statistically significant.

## Results

### Performance of LISA for the diagnosis of syphilis

**Figure 2.**
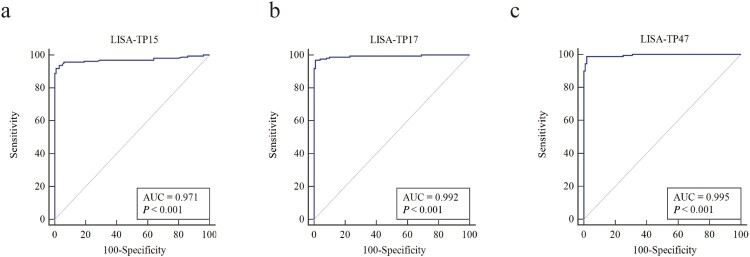
ROC analysis of the diagnostic performance of LISA-TP15, LISA-TP17, and LISA-TP47 in 261 participants. The AUC of both LISA-TP17 and LISA-TP47 was significantly higher than that of LISA-TP15, as determined by the DeLong test. Abbreviations: ROC, Receiver Operating Characteristic; LISA, luciferase immunosorbent assay; AUC, Area Under Curve.

**Figure 3.**
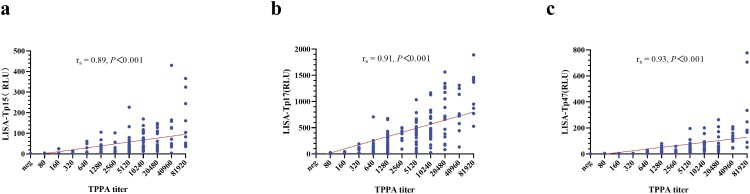
Correlation between serum TPPA titres and LISA-TP15, LISA-TP17, and LISA-TP47 in 258 participants. Three of 261 serum samples used for the evaluation of LISA’s diagnostic performance were excluded from this analysis as they lacked TPPA results with the maximum dilution. Abbreviations: TPPA, *Treponema pallidum* particle agglutination; LISA, luciferase immunosorbent assay; RLU, relative light unit; neg, negative.

A total of 261 serum samples (Supplemental Table 1) were used for evaluating the diagnostic performance of three LISA tests (LISA-TP15, LISA-TP17, and LISA-TP47) by using TPPA as a reference (Table 1). LISA-TP17 showed a sensitivity of 96.9% and specificity of 99.0% while +LR and −LR were 96.9 and 0.031. The corresponding values for LISA-TP47 were 98.8%, 98.0%, 49.4, and 0.013, respectively (Table 1). For 19 samples with discordant results between LISA and TPPA, further validation with FTA-Abs and CLIA indicated that 15 were positive (Supplemental Table 2). Furthermore, all three LISA tests demonstrated excellent performance in the ROC analysis with an AUC greater than 0.95. The AUC of both LISA-TP17 (0.992, 95% CI: 0.973–0.999) and LISA-TP47 (0.995, 95% CI: 0.977–1.000) was significantly better than LISA-TP15 (0.971, 95% CI: 0.942–0.988) with a *P*-value of .015 and .023, respectively (Figure 2). When the S/CO ratio was 2 or greater, the specificity of LISA-TP15, LISA-TP17, and LISA-TP47 could reach 100% while the sensitivity remained as high as 90% (Supplemental Table 3). In addition, the improved performance of the combined test using LISA-TP15, LISA-TP17, and LISA-TP47 were illustrated (Table 1). The correlation between RLU levels of LISA and TPPA titres was evaluated in 258 subjects using Spearman’s rank correlation and showed a strong correlation between TPPA titres and the RLU levels of LISA-TP15 (*r_s_* = 0.89, *P *< .0001), LISA-TP17 (*r_s_* = 0.91, *P *< .0001), and LISA-TP47 (*r_s_* = 0.93, *P *< .0001) (Figure 3).

**Table 1.** Diagnostic efficacy of LISA-TP15, LISA-TP17, LISA-TP47 and their combined tests in syphilis diagnosis.

Assays	TPPA result (*n* = 261)		Sensitivity(%, 95% CI)	Specificity(%, 95% CI)	+LR(95% CI)	−LR(95% CI)
	Positive(*n* = 161)	Negative(*n* = 100)				
LISA-TP15						
Positive	148	1	91.9 (86.6–95.6)	99.0 (94.6–100.0)	91.9 (13.1–646.6)	0.082 (0.05–0.1)
Negative	13	99				
LISA-TP17						
Positive	156	1	96.9 (92.9–99.0)	99.0 (94.6–100.0)	96.9 (13.8–681.3)	0.031 (0.01–0.07)
Negative	5	99				
LISA-TP47						
Positive	159	2	98.8 (95.6–99.8)	98.0 (93.0–99.8)	49.4 (12.5–194.7)	0.013 (0.003–0.05)
Negative	2	98				
LISA-TP15 + LISA-TP17^a^						
Positive	157	2	97.5 (93.8–99.3)	98.0 (93.0–99.8)	48.76 (12.4–192.3)	0.025 (0.010–0.07)
Negative	4	98				
LISA-TP15 + LISA-TP47^a^						
Positive	160	3	99.4 (96.6–100.0)	97.0 (91.5–99.4)	33.13 (10.9–101.0)	0.0064 (0.0009–0.05)
Negative	1	97				
LISA-TP17 + LISA-TP47^a^						
Positive	160	3	99.4 (96.6–100.0)	97.0 (91.5–99.4)	33.13 (10.9–101.0)	0.0064 (0.0009–0.05)
Negative	1	97				
LISA-TP15 + LISA-TP17 + LISA-TP47^a^						
Positive	160	4	99.4 (96.6–100.0)	96.0 (90.1–98.9)	24.84 (9.5–64.9)	0.0065 (0.0009–0.05)
Negative	1	96				

Notes: In this study, 261 subjects were evaluated. A TPPA titre of ≥1:80 was considered positive. The optimal cut-off values for LISA-TP15, LISA-TP17, and LISA-TP47 were determined using the Receiver Operating Characteristic curve. In most circumstances, assays with +LR ≥ 10 are considered to provide strong evidence to rule in diagnosis, and assays with −LR ≤ 0.1 are considered to provide strong evidence to rule out diagnosis.

Abbreviations: LISA, luciferase immunosorbent assay; TPPA, *Treponema pallidum* particle agglutination; +LR, positive likelihood ratio; −LR, negative likelihood ratio; CI, confidence interval.

^a^ Combined tests were considered positive if any of the tests were positive, negative otherwise.

We also compared the levels of serum antibodies against TP15, TP17, and TP47 in the patients at different stages of syphilis and found that they were relatively low in the patients of primary syphilis while high levels of antibodies against TP15, TP17, and TP47 were observed in the patients of secondary syphilis, early latent syphilis, and late latent syphilis, although subtle differences existed (Supplemental Figure 2).

### Dynamic profile of anti-syphilis antibodies over time in T. pallidum-infected rabbits

**Figure 4.**
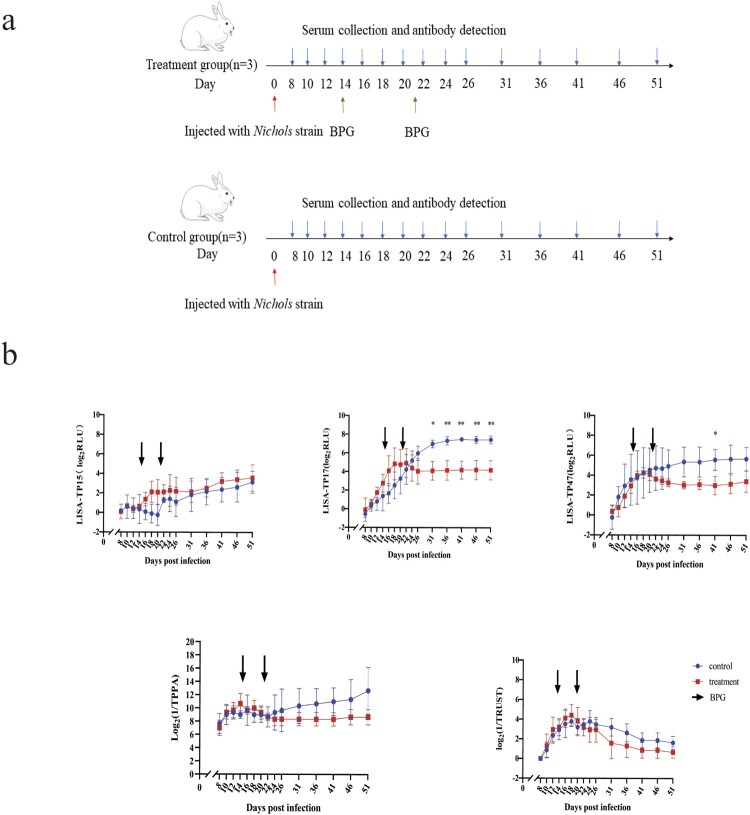
Antibody titres profile of LISA-TP15, LISA-TP17, LISA-TP47, TPPA, and TRUST in *T. pallidum*-infected rabbits. (a) Experiment with *Nichols* strain infection, BPG therapy and serum collection in New Zealand white Rabbits. The treatment group received intramuscular administration of 200,000 units of BPG 14 and 21 days post-infection. (b) Anti-syphilis antibody profile in New Zealand white rabbits. The log-transformed data were presented as mean ± standard deviation. Differences in antibody levels between the two groups were compared using a Student’s *t*-test. *P < .05, **P < .01. Abbreviations: LISA, luciferase immunosorbent assay; TPPA, *Treponema pallidum* particle agglutination; TRUST, toluidine red unheated serum test; BPG, benzathine penicillin; RLU, relative light unit.

Figure 4 depicts the change in treponemal antibody and non-treponemal antibody titres in the serum of *T. pallidum*-infected New Zealand white rabbits before and after treatment with BPG. As seen in the treatment group, the levels of treponemal antibodies tested by LISA-TP17, LISA-TP47, and TPPA gradually increased and reached the peak after *T. pallidum* infection, but declined and eventually stabilized after BPG treatment. However, the level of treponemal antibody tested by LISA-TP15 in the treatment group showed an increasing trend post-BPG treatment. The TRUST titres increased followed by decreasing or becoming undetectable in both the treatment and control groups. Serological reactivity to *T. pallidum* infection in five tests in the treatment group and control group were depicted in Supplemental Figure 1. There was no significant difference between the two groups in terms of the antibody levels of LISA-TP15, TPPA, and TRUST. For LISA-TP17, there was no statistical significance between the treatment and control groups for the first 26 days post-infection, but a significant difference was observed 31–51 days post-infection. For LISA-TP47, a significant difference was only seen 41 days post-infection.

### Decline of serum TP15, TP17, and TP47 antibody levels and TRUST titres after treatment in patients with syphilis of different stages

**Figure 5.**
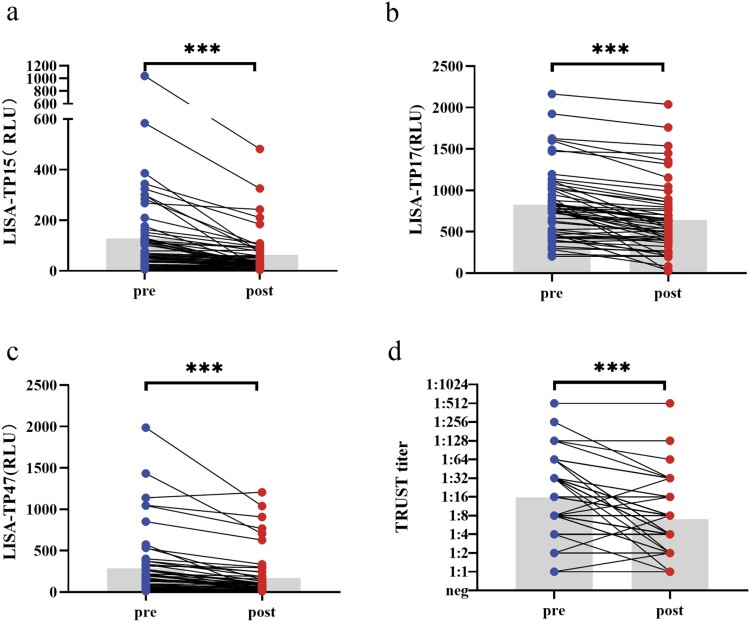
Changes in antibody responses in paired pre- and post-treatment serum samples from 55 syphilis patients. Change of TPPA titres pre- and post-treatment was unavailable for the lack of TPPA results with the maximum dilution. Wilcoxon matched-pairs signed rank test was used to test for differences in antibody levels pre- and post-treatment. ***P < .001. Abbreviations: RLU, relative light unit; neg, negative.

A total of 110 paired follow-up samples were collected from patients with different stages of syphilis, including 9 early syphilis, 9 late syphilis, 10 probable NS, and 27 confirmed NS (Supplemental Table 4). The results showed that the level of serum antibodies against TP15, TP17, and TP47 tested by LISA and TRUST were significantly higher before treatment than after treatment (Figure 5, *P *< .001). The treatment dramatically inhibited the serum antibody response of anti-TP15, TP17, TP47 and TRUST titres tested in the patients of early syphilis and confirmed NS while only TP17 and TP47 antibodies significantly decreased in the patients of late syphilis and probable NS (Supplemental Table 5). For the patients treated with benzathine penicillin G/aqueous crystalline penicillin G, the level of serum antibodies against TP15, TP17, TP47 and TRUST titres decreased significantly after treatment. However, for the patients treated with ceftriaxone, a significant difference was only observed in the change of the level of serum antibodies against TP15 and TRUST titres after treatment (Supplemental Table 6). The Hodges–Lehmann estimate revealed a significant decline in the TRUST titres and in the level of serum antibodies against TP15, TP17, and TP47 tested by LISA after treatment (Table 2). For the participants whose TRUST titres didn’t change, or decreased by 2-fold, 4-fold or ≥8-fold after treatment, the corresponding reduction of antibody levels was 17.53%, 31.34%, 48.62%, and 72.79% for LISA-TP15, respectively; 8.84%, 17.00%, 28.37%, and 50.57% for LISA-TP17, respectively; and 22.25%, 29.79%, 51.75%, and 70.28% for LISA-TP47, respectively. Compared to the patients with a decrease of <48.62%, 28.37%, 51.75% after treatment in anti-TP15, anti-TP17, anti-TP47 antibodies, respectively, the patients with a decrease of ≥48.62%, 28.37%, 51.75% in anti-TP15, anti-TP17, anti-TP47 antibodies, respectively, were more likely to have a decrease of ≥4-fold in TRUST titres (Supplemental Table 7). In addition, similar correlation was found between the change of TRUST titres and the decline of antibody levels for LISA-TP15 (*r_s_* = 0.63, *P *< .001), LISA-TP17 (*r_s_* = 0.61, *P *< .001), and LISA-TP47 (*r_s_* = 0.63, *P *< .001, Table 3).

**Table 2.** Assessing median changes in serum antibody levels of LISA-TP15, LIAS-TP17, LISA-TP47, based on TRUST titres changes post-syphilis treatment in 51 participants^a^.

*X* fold decline in TRUST titres (*n*)	LISA-TP15 median percent changes (%)		LISA-TP17 median percent changes (%)		LISA-TP47 median percent changes (%)	
	Point estimate	(95% CI)	Point estimate	(95% CI)	Point estimate	(95% CI)
*X* = 0 (17)	−17.53	(−29.76, −7.57)	−8.84	(−15.41, −2.99)	−22.25	(−33.99, −12.10)
*X* = 2 (16)	−31.34	(−42.62, −22.61)	−17.00	(−23.41, −11.66)	−29.79	(−46.94, −18.63)
*X* = 4 (8)	−48.62	(−73.26, −23.32)	−28.37	(−48.35, −8.96)	−51.75	(−72.38, −29.62)
*X* ≥ 8 (10)	−72.79	(−85.99, −60.39)	−50.57	(−70.56, −33.77)	−70.28	(−83.80, −57.61)

Notes: Median percent changes, along with 95% CI, of antibodies against TP15, TP17, and TP47 measured by LISA in longitudinal serum samples were calculated using the Hodges–Lehmann estimate, stratified by TRUST titres decline. A 0, 2, 4, and 8-fold change in the TRUST titres represented a 0-fold (no change), a 2-fold (one dilution), a 4-fold (two dilution), and a 8-fold (three dilution) change, respectively.

Abbreviations: TRUST, toluidine red unheated serum test; CI, confidence interval; LISA, luciferase immunosorbent assay.

^a^ The analysis included 102 paired follow-up samples from 17 non-neurosyphilis patients and 34 neurosyphilis patients. Four patients who showed a 2- or 4-fold increase in TRUST titres post-treatment were excluded due to a small sample size.

**Table 3.** Correlation between changes in serum antibody levels against TP15, TP17, and TP47 tested by LISA and serum TRUST titres after syphilis treatment in 55 participants.

LISA	Baseline TRUST titres		TRUST titres change	
	*r_s_*	*P-*value	*r_s_*	*P-*value
LISA-TP15	0.44	<.001	0.63	<.001
LISA-TP17	0.33	.01	0.61	<.001
LISA-TP47	0.53	<.001	0.63	<.001

Notes: Analysis of correlation used Spearman’s rank correlation.

Abbreviations: LISA, luciferase immunosorbent assay; TRUST, toluidine red unheated serum test.

## Discussion

In this study, we developed LISA to detect specific IgG against TP15, TP17 and TP47, and evaluated its performance in diagnosing syphilis and monitoring the change of syphilis antibody levels after treatment in both *T. pallidum*-infected rabbit model and syphilis patients.

The three *T. pallidum*-specific antigens, TP15, TP17, and TP47, showed excellent performance (AUC > 0.95) in syphilis diagnosis in our LISA platform. LISA-TP17 and LISA-TP47 demonstrated significantly higher sensitivity than LISA-TP15, suggesting the great potential of LISA-TP17 and LISA-TP47 in syphilis diagnosis. Similar results have been reported in several studies [21,22]. For example, an in-house whole-cell lysate antigen-based western immunoblotting (wclWB) showed that 99.5%, 89.0%, and 77.5% of the specimens from syphilis patients were reactive with antigen TP47, TP17, and TP15, respectively [21]. In another study, 100% (20/20), 75% (15/20), and 25% (5/20) of primary syphilis patients reacted with antigen TP47, TP17 and TP15, respectively using Western blotting (WB) [22]. Compared with the classic TPPA assay, LISA is more accurate and objective [19,20]. Therefore, LISA, especially LISA-TP17 and LISA-TP47, may be a suitable alternative to TPPA for the diagnosis of syphilis. In addition, our results show the different levels of anti-*T. pallidum* antibodies at different stages of syphilis patients, suggesting that the level of serum antibodies against TP15, TP17, and TP47 may be used for aid in distinguishing patients at different stages of syphilis.

We would like to emphasize that the aim of our study was to evaluate the performance and feasibility of different *T. pallidum*-specific antigens in our LISA platform. Therefore, LISA-TP15, LISA-TP17, LISA-TP47 were separately developed and evaluated. Although all three methods demonstrated excellent performance in the diagnosis of syphilis and in the evaluation of treatment efficacy, subtle differences were observed. In general, LISA-TP17 and LISA-TP47 were better than LISA-TP15. In addition, we found that the combination of LISA-TP15, LISA-TP17 and LISA-TP47 yielded the highest sensitivity of 99.4% and specificity of 96.0% (Table 1). These findings provide critical data for developing a methodology based on LISA with the combination of TP15, TP17, and TP47 recombinant antigens.

Furthermore, LISA as a tool to monitor syphilis treatment efficacy relies on its broader linear range, which has been characterized in the previous study [10] and confirmed in the current study. LISA showed a wide linear range of detection from 10 pg/mL to 100 ng/mL and is 10^4^-fold more sensitive than ELISA when detecting anti-HIV-1 p24 antibody [10], and has been used to monitor the antiretroviral treatment (ART) efficacy in HIV-infected patients [11]. We found that ART led to the reduction of anti-HIV antibodies by more than 75% of the baseline levels 24 months post-treatment in 52% of chronically HIV-infected patients. In addition, Burbelo et al. reported a similar luciferase immunoprecipitation systems (LIPS), which shows higher sensitivity and larger dynamic range over existing standard assays for measuring antibodies in autoimmune and infectious diseases [23]. Moreover, in the current study, a robust correlation was observed between the antibody levels of TP15, TP17, and TP47 tested by LISA and TPPA titres (correlation coefficient 0.89–0.93, *P *< .001), indicating that LISA could provide a semi-quantitative estimate of syphilis antibodies. These results demonstrate that LISA as a more sensitive assay with a wider range of detection of antibodies could be used for monitoring treatment efficacy.

In clinical practice, NTTs, such as RPR/VDRL/TRUST, are commonly used to evaluate the efficacy of treatment for syphilis [3,8]. However, their usefulness is limited by the potential of false-positive results and serofast [4,5,18]. Therefore, the detection of *T. pallidum*-specific antibodies for evaluating the efficacy of treatment in clinical settings is a new field of research [9,24–27]. However, there are inconsistent results about the use of *T. pallidum-*specific antibodies for assessing the effectiveness of therapy. Several studies have shown that treponemal antibodies persist in syphilis patients and are traditionally considered to be unrelated to disease activity [8,18]. Maciej Pastuszczak and colleagues [24] found that the reactivity to the treponemal antigens TP15, TP17, TP47, and TmpA did not change one year after treatment using the INNO-LIA Syphilis Score Assay. Liu and colleagues [25] also found no significant statistical difference (*P* > .05) in the S/CO values of TP-ELISA and TP-CMIA in 30 syphilis patients before and after three months of treatment. In contrast, several studies support the use of treponemal antibody as a reliable indicator of treatment efficacy [26,27]. One study showed that the level of TmpA antibody tested via ELISA significantly decreased within a year of antibiotic treatment in syphilis patients [26]. Another study found that changes in the antibody levels of phase-dependent Tp0971 and TmpA, tested by ELISA, were positively correlated with changes in the serum RPR titres before and after syphilis treatment [27]. These research results suggest that treatment for syphilis may lead to a reduction in the antibody against certain *T. pallidum* antigens, such as Tp0971 and TmpA.

In our study, we found that the antibodies against TP15, TP17, and TP47 tested by LISA may be useful in evaluating the effectiveness of syphilis treatment. In the rabbit infection models, we observed that the levels of treponemal antibodies as determined by LISA-TP17, LISA-TP47, and TPPA declined after treatment with BPG, and eventually stabilized. Consistent with a previous study by Lin et al. [28], in our study TRUST titres increased and then decreased (even became negative) in the control group, similar to that seen in the treatment group. Similarly, it was found that non-treponemal test titres often decline rapidly post-treatment in the syphilis patients although decreased more slowly in the absence of treatment [29]. Furthermore, we observed a correlation between the percentage change of antibodies against TP15, TP17, and TP47 and the change in TRUST titres before and after treatment in syphilis patients. Previous studies have revealed effective treatment when antibody titres decline by ≥4 folds (two dilutions e.g. 1:16–1:4) in NTTs or become sero-negative after therapy [8]. In our study, 32.7% (18/55) of syphilis patients for assessing treatment response reached a 4-fold decrease in TRUST titres after treatment. Participants who showed a 4-fold decrease in TRUST titres after treatment had a median reduction of 48.62%, 28.37%, and 51.75% in the levels of TP15, TP17, and TP47 antibodies, respectively. These results suggest that the *T. pallidum*-specific antibody levels could potentially be used for evaluating treatment efficacy. In fact, Hu et al. reported that BPG treatment resulted in ≥4-fold decrease in TPPA titres [30]. They found that 99% (109/110) of patients with TPPA titres decreased by more than 4-fold were serological cure patients. In addition, the TPPA titres significantly decreased in *T. pallidum*-infected and treated rabbits, but not in the un-treatment rabbits. Additionally, Sun et al. found that the levels of anti-TP17 IgG and anti-TP47 IgG tested by Western blotting decreased following anti-syphilis treatment [31]. These results support that the decrease in antibody response against *T. pallidum* antigens, such as TP17 and TP47, may indicate effective treatment in syphilis. Our findings are consistent with those previous studies [30,31]. Like ART to inhibit HIV-1 replication and regulate antibody response in HIV-infected patients, syphilis treatment may alleviate the antibody response against *T. pallidum* antigens, such as TP17 and TP47 [9]. However, in a study involving children with confirmed yaws caused by *T. pallidum* subspecies *pertenue* [32], no significant decrease of anti-TP17 antibodies was observed post-treatment. Further studies are warranted to elucidate the reason for the differential antibody response between the homologous proteins of *T. pallidum* subspecies *pertenue* and *T. pallidum* ssp. *pallidum*.

Our study also has some limitations. Firstly, the performance of LISA in syphilis diagnosis and treatment efficacy monitoring was not explicitly explored in special syphilis cases, such as syphilis during pregnancy and congenital syphilis. Further research needs to encompass a broader range of syphilis cases to validate the value of serum antibodies against TP15, TP17, and TP47 in both syphilis diagnosis and treatment efficacy monitoring. Secondly, in the rabbit infection model, a duplicate test was not performed due to the scarcity of biological samples. However, the results of the rabbit infection model provided valuable insights for future exploration of the profile of treponemal antibodies before and after treatment. Thirdly, in the rabbit infection model, a longer observation period is needed to better understand the dynamic profile of antibodies over time although a relatively short follow-up time of six weeks was used to evaluate treatment efficacy in the rabbit models [33,34]. Fourthly, in paired samples from syphilis patients, the relatively short follow-up period may affect the determination of serological cure in patients with less than a 4-fold change in TRUST titres after treatment. Therefore, a longer follow-up period would be necessary to accurately evaluate the relationship between changes in treponemal antibodies and treatment efficacy.

In summary, our research indicates that LISA yielded comparable results to the TPPA assay, in particular, LISA-TP17 and LISA-TP47 exhibiting exceptional diagnostic accuracy. Additionally, LISA offers the advantage of semi-quantitative measurement of syphilis antibodies, which may be useful in monitoring the antibody levels against TP15, TP17, and TP47 and in evaluating the efficacy of syphilis treatment.

## Supplementary Material

Supplemental_material_original

## Data Availability

A valid and legitimate reason to request access to relevant original data from the corresponding author.
