# Supplementary material for: Evaluating the clinical utility of semi-quantitative luciferase immunosorbent assay using Treponema pallidum antigens in syphilis diagnosis and treatment monitoring
Source: Emerg Microbes Infect. 2024 Apr 25;13(1):2348525. doi: 10.1080/22221751.2024.2348525 (PMC11100446; doi:10.1080/22221751.2024.2348525)
Supplement: Supplemental_material_original [file TEMI_A_2348525_SM6964.docx]

**Supplemental Table 1. Demographic and Clinical Characteristics of 201 STD Clinic Patients for Evaluating LISA**

| **Characteristic** | **Participant (n=201)** |
| --- | --- |
| Gender, n (%) ^a^ |  |
| Male | 139 (69.2) |
| Female | 62 (30.8) |
| Age, median (IQR), years ^a^ | 31 (25-45) |
| Clinical diagnosis, n (%) ^a^ |  |
| Primary syphilis | 11 (5.5) |
| Secondly syphilis | 26 (12.9) |
| Early latent syphilis | 18 (9.0) |
| Late latent syphilis | 11 (5.5) |
| Latent syphilis of unknown duration | 94 (46.8) |
| Non-syphilis | 33 (16.4) |
| TPPA results, n (%) |  |
| Negative (-) | 40 (19.9%) |
| Positive (+) | 161 (80.1%) |
| TPPA titers, median (IQR) ^b^ | 1:1280 (1:160-1:10240) |
| TRUST results, n (%) ^c^ |  |
| Negative (-) | 69 (34.3%) |
| Positive (+) | 127 (63.2%) |
| TRUST titers, median (IQR) ^c^ | 1:2 (negative-1:8) |

^a^ Among 201 patients from the STD clinic, 160 were diagnosed with syphilis and 33 with non-syphilis. Data of clinical diagnosis for 8 participants were incomplete. 60 TPPA-negative healthy blood donors were not included due to lack of demographic data.

^b^ 3 participants did not have TPPA titer results.

^c^ 5 participants did not have TRUST results.

Abbreviations: LISA, luciferase immunosorbent assay; IQR, interquartile range; TPPA, *Treponema pallidum* particle agglutination; TRUST, toluidine red unheated serum test.

**Supplemental Table 2. Results of Treponemal Tests for the Samples with Discordant Results between LISA and TPPA Assay.**

| **No** | **TPPA** | **CLIA** | **FTA-Abs** | **Consensus results^a^** |
| --- | --- | --- | --- | --- |
| 1 | + | + | + | + |
| 2 | + | + | + | + |
| 3 | + | + | + | + |
| 4 | + | + | + | + |
| 5 | + | + | + | + |
| 6 | + | + | + | + |
| 7 | + | + | + | + |
| 8 | + | - | + | + |
| 9 | + | + | + | + |
| 10 | + | + | + | + |
| 11 | + | - | + | + |
| 12 | + | + | + | + |
| 13 | + | - | + | + |
| 14 | + | + | + | + |
| 15 | + | + | + | + |
| 16 | - | - | - | - |
| 17 | - | + | - | - |
| 18 | - | + | - | - |
| 19 | - | - | - | - |

^a^ Consensus results mean positive in at least 2 out of 3 treponemal tests (TPPA, CILA, and FTA-Abs).

Abbreviations: TPPA, Treponema pallidum particle agglutination; CLIA, chemiluminescence immunoassays; FTA-Abs, fluorescent treponemal antibody absorption.

**Supplemental Table 3. Diagnostic Accuracy of LISA-TP15, LISA-TP17, and LISA-TP47 in Syphilis Diagnosis in 261 Participants according to S/CO values**

| **S/CO** | **LISA-TP15** | | | |  | **LISA-TP17** | | | |  | **LISA-TP47** | | | |
| --- | --- | --- | --- | --- | --- | --- | --- | --- | --- | --- | --- | --- | --- | --- |
|  | **TPPA +** | **TPPA -** | **Sensitivity**  **(%, 95% CI)** | **Specificity**  **(%, 95% CI)** |  | **TPPA +** | **TPPA -** | **Sensitivity**  **(%, 95% CI)** | **Specificity**  **(%, 95% CI)** |  | **TPPA +** | **TPPA -** | **Sensitivity**  **(%, 95% CI)** | **Specificity**  **(%, 95% CI)** |
| ≤0.5 | 7 | 83 | 95.7  (91.2-98.2) | 83.0  (74.2-89.8) |  | 1 | 54 | 99.4  (96.6-100.0) | 54.0  (43.7-64.0) |  | 1 | 72 | 99.4  (96.6-100.0) | 72.0  (62.1-80.5) |
| ＞0.5 | 154 | 17 |  |  |  | 160 | 46 |  |  |  | 160 | 28 |  |  |
| ≤1 | 13 | 99 | 91.9  (86.6-95.6) | 99.0  (94.6-100.0) |  | 5 | 99 | 96.9  (92.9-99.0) | 99.0  (94.6-100.0) |  | 2 | 98 | 98.8  (95.6-99.8) | 98.0  (93.0-99.8) |
| ＞1 | 148 | 1 |  |  |  | 156 | 1 |  |  |  | 159 | 2 |  |  |
| ≤1.5 | 20 | 100 | 87.6  (81.5-92.2) | 100.0  (96.4-100.0) |  | 10 | 99 | 93.8  (88.9-97.0) | 99.0  (94.6-100.0) |  | 9 | 99 | 94.4  (89.7-97.4) | 99.0  (94.6-100.0) |
| ＞1.5 | 141 | 0 |  |  |  | 151 | 1 |  |  |  | 152 | 1 |  |  |
| ≤2 | 29 | 100 | 82.0  (75.2-87.6) | 100.0  (96.4-100.0) |  | 13 | 100 | 91.9  (86.6-95.6) | 100.0  (96.4-100.0) |  | 16 | 100 | 90.1  (84.4-94.2) | 100.0  (96.4-100.0) |
| ＞2 | 132 | 0 |  |  |  | 148 | 0 |  |  |  | 145 | 0 |  |  |

Abbreviations: LISA, luciferase immunosorbent assay; S/CO, signal-to-cutoff; TPPA, Treponema pallidum particle agglutination; CI, confidence interval.

**Supplemental Table 4. Demographic and Clinical Characteristics of 55 Patients at Different Syphilis Stages for Assessing Treatment Response**

| **Characteristic** | **Syphilis (n=55)** |
| --- | --- |
| Gender, n (%) |  |
| Male | 32 (58.2) |
| Female | 23 (41.8) |
| Age [median (IQR), years] | 37 (27-54) |
| TRUST titer before treatment, median (IQR) | 1:16 (1:8-1:32) |
| TRUST titer after treatment, median (IQR) | 1:8 (1:4-1:16) |
| Clinical diagnosis, n (%) ^a^ |  |
| Primary syphilis | 1 (1.8) |
| Secondary syphilis | 4 (7.3) |
| Latent syphilis | 13 (23.6) |
| Confirmed NS | 27 (49.1) |
| Probable NS | 10 (18.2) |
| Therapy, n (%) |  |
| Benzathine penicillin G^b^ | 18 (32.7) |
| Ceftriaxone^c^ | 9 (16.4) |
| Aqueous crystalline penicillin G^c^ | 28 (50.9) |
| Time interval between two follow-up visits, median (IQR), months | 6 (6-12) |

^a^ The diagnosis of primary, secondary, latent syphilis was based on the guideline of the STD Association, China CDC [1]; NS diagnosis was based on the guidelines from UpToDate (2020) [2], the Centers for Disease Control and Prevention (CDC) of the USA (2021) [3], and Europe (2020) [4];

^b^ Primary/secondary/latent late syphilis patients were all initially diagnosed, and received BPG therapy (2.4 million units weekly for 2/3 consecutive weeks) after enrollment, and follow-up visits were conducted at least three months apart;

^c^ NS patients received intravenous aqueous crystalline penicillin G (3–4 million units every 4 hours for 14 days) or intravenous ceftriaxone (2 g once daily 14 days) after enrollment, and follow-up visits were conducted at least six months apart;

Abbreviations: TRUST, toluidine red unheated serum test; IQR, interquartile range; NS, neurosyphilis.

| **Assays** | **Early syphilis ^a^ (n=9)** | | ***P*** | **Late syphilis ^b^ (n=9)** | | ***P*** | **Probable NS (n=10)** | | *P* | **Confirmed NS (n=27)** | | ***P*** |
| --- | --- | --- | --- | --- | --- | --- | --- | --- | --- | --- | --- | --- |
|  | **pre-treatment** | **post-treatment** |  | **pre-treatment** | **post-treatment** |  | **pre-treatment** | **post-treatment** |  | **Pre-treatment** | **post-treatment** |  |
| LISA-TP15  (Median [IQR]) | 116.1 [38.6;120.1] | 17.0 [10.0;32.0] | 0.008 | 36.0 [17.7;54.4] | 37.0 [17.0;43.0] | 0.086 | 66.6 [36.3;177.4] | 46.0 [25.0;75.8] | 0.028 | 102.2 [46.5;158.4] | 38.0 [21.5;85.0] | <0.001 |
| LISA-TP17  (Median [IQR]) | 629.0 [468.0;750.0] | 322.0 [192.0;490.0] | 0.008 | 431.0 [297.0;662.0] | 399.0 [270.0;634.0] | 0.012 | 995.0 [481.2;1380.8] | 703.5 [430.8;1211.2] | 0.047 | 825.0 [742.0;1075.0] | 667.0 [490.5;828.0] | <0.001 |
| LISA-TP47  (Median [IQR]) | 154.0 [51.0;220.0] | 25.0 [12.0;41.0] | 0.012 | 29.0 [26.0;76.0] | 26.0 [20.0;50.0] | 0.013 | 90.0 [49.8;159.8] | 59.0 [33.0;102.2] | 0.005 | 261.0 [134.0;553.5] | 107.0 [48.0;314.5] | <0.001 |
| TRUST  (Median [IQR]) | 1:32  [1:8; 1:32] | 1:4  [1:2; 1:4] | 0.018 | 1:4  [1:2; 1:8] | 1:4  [1:2; 1:4] | 0.285 | 1:8  [1:8; 1:8] | 1:4  [1:4; 1:8] | 0.173 | 1:32  [1:8; 1:64] | 1:8  [1:8; 1:32] | <0.001 |

**Supplemental Table 5. Changes in Antibody** **Responses to TP15, TP17 and TP47 Tested by LISA and TRUST in Paired Pre- and Post-Treatment Serum Samples, Stratified by Syphilis Stage**

Wilcoxon matched-pairs signed rank test was used to test for differences in antibody levels pre- and post- treatment.

^a^ Early syphilis included primary, secondary, and early latent syphilis; ^b^ Late syphilis included late latent syphilis and latent syphilis of unknown duration.

Abbreviations: NS, neurosyphilis; LISA, luciferase immunosorbent assay; IQR, interquartile range; TRUST, toluidine red unheated serum test.

**Supplemental Table 6. Changes in Antibody Responses to TP15, TP17 and TP47 Tested by LISA and TRUST in Paired Pre- and Post-Treatment Serum Samples, Stratified by Syphilis Therapy**

| **Assays** | **Benzathine penicillin G (n=18)** | | ***P*** | **Ceftriaxone (n=9)** | | ***P*** | **Crystalline penicillin G (n=28)** | | ***P*** |
| --- | --- | --- | --- | --- | --- | --- | --- | --- | --- |
|  | **pre-treatment** | **post-treatment** |  | **pre-treatment** | **post-treatment** |  | **pre-treatment** | **post-treatment** |  |
| LISA-TP15  (Median [IQR]) | 116.1 [38.6;120.1] | 17.0  [10.0;32.0] | 0.001 | 60.4 [51.4;268.3] | 33.0  [23.0;55.0] | 0.011 | 90.5  [39.1;155.8] | 40.0  [21.8;84.5] | <0.001 |
| LISA-TP17  (Median [IQR]) | 629.0 [468.0;750.0] | 322.0 [192.0;490.0] | <0.001 | 775.0 [527.0;1008.0] | 545.0 [403.0;704.0] | 0.051 | 869.0 [744.5;1204.2] | 700.0 [509.2;958.8] | <0.001 |
| LISA-TP47  (Median [IQR]) | 154.0 [51.0;220.0] | 25.0  [12.0;41.0] | <0.001 | 129.0 [97.0;314.0] | 105.0 [60.0;295.0] | 0.065 | 210.0 [93.0;433.0] | 96.5  [41.0;240.5] | <0.001 |
| TRUST  (Median [IQR]) | 1:32 [1:8; 1:32] | 1:4 [1:2; 1:4] | 0.007 | 1:16 [1:8;1:32] | 1:8 [1:4;1:8] | 0.018 | 1:32 [1:8;1:64] | 1:8 [1:4;1:16 ] | 0.001 |

The Wilcoxon matched-pairs signed rank test was employed to assess differences in antibody levels pre and post treatment.

Abbreviations: LISA, luciferase immunosorbent assay; IQR, interquartile range; TRUST, toluidine red unheated serum test.

**Supplemental Table 7. Comparison of Changes in Antibody Responses to TP15, TP17 and TP47 Tested by LISA between Participants with≥4-fold or ＜4-fold Decrease in Serum TRUST Titers after Treatment**

| **Characteristic** | **≥4-fold decrease in TRUST titers (%)** | **＜4-fold decrease in TRUST titers (%)** | ***P*** |
| --- | --- | --- | --- |
| Decrease in anti-TP15 antibodies |  |  | <0.001 |
| ≥ 48.62% | 14 (77.8) | 4 (22.2) |  |
| ＜ 48.62% | 4 (12.1) | 29 (87.9) |  |
| Decrease in anti-TP17 antibodies |  |  | <0.001 |
| ≥ 28.37% | 10 (76.9) | 3 (23.1) |  |
| ＜ 28.37% | 8 (21.1) | 30 (78.9) |  |
| Decrease in anti-TP47 antibodies |  |  | <0.001 |
| ≥ 51.75% | 13 (72.2) | 5 (27.8) |  |
| ＜ 51.75% | 5 (15.2) | 28 (84.8) |  |

The analysis included 102 paired follow-up samples from 17 non-neurosyphilis patients and 34 neurosyphilis patients. Four patients who showed a 2- or 4-fold increase in TRUST titers post-treatment were excluded due to a small sample size.

Chi-square test was conducted to compare changes in antibody responses to TP15, TP17 and TP47 tested by LISA between participants with≥4-fold or ＜4-fold decrease in serum TRUST titers after treatment.

Abbreviations: LISA, luciferase immunosorbent assay; TRUST, toluidine red unheated serum test.


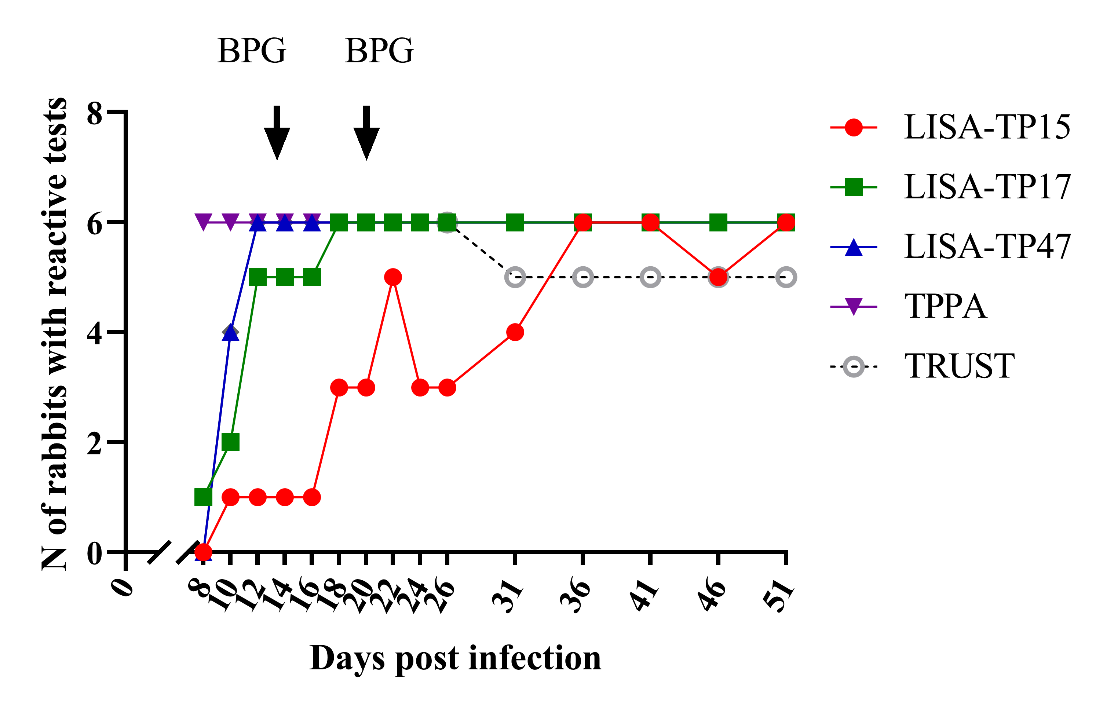


**Supplemental Figure 1. Serological Reactivities to Treponema pallidum Infection in Treated and Untreated New Zealand White Rabbits.**

In this study, the *Nichols* strain of *Tp* was propagated in male New Zealand white rabbits (3 months old and weighing 3 kg) through intratesticular inoculation. Before infection, each rabbit was tested for *T. paraluiscuniculi* infection using TPPA and TRUST [5, 6]. Only rabbits seronegative by both TPPA and TRUST tests were used for *T. pallidum* propagation. The rabbits were then inoculated with 2 mL of treponemal suspension and maintained at 18℃. The testicles of infected rabbits were removed when orchitis appeared and serum samples were tested positive for TPPA. After collecting the treponemal suspension, 2 mL of the suspension were re-injected into the testis of the new rabbits and the related samples were harvested using the same method described above. After harvesting the treponemal suspension, six male New Zealand white rabbits (3 months old and weighing 3 kg, with negative results for TPPA and TRUST tests) were injected intradermally at 10 sites on their backs with 0.1 mL (10^7^/mL) treponemal suspension. Three of these rabbits in the treatment group were then treated with intramuscular injections of 200,000 U of BPG on the 14^th^ day and 21^th^ day post-infection. Challenge experiment with the *Nichols* strain of *T*p and antimicrobial therapy were previously described [7-9]. Serum samples were collected from each rabbit at regular intervals, every 2 days from day 8 to 26 post-infection, and every 5 days from day 26 to 51 post-infection for TRUST, TPPA, LISA-TP15, LISA-TP17, and LISA-TP47 tests. A TPPA titer of ≥1:80 was considered positive. The optimal cut-off values for LISA-TP15, LISA-TP17, and LISA-TP47 were determined using the Receiver Operating Characteristic Curve analysis (Table 1). Seroconversion of LISA-TP15, LISA-TP17, LISA-TP47, TPPA, and TRUST in six New Zealand white rabbits started from the 10^th^,8^th^,10^th^,8^th^, and 10^th^ days, respectively; results for LISA-TP15, LISA-TP17, LISA-TP47, TPPA, and TRUST were all positive in the six New Zealand white rabbits on the 36^th^, 18^th^, 12^th^, 8^th^, and 12^th^ days, respectively. The results of LISA-TP17, LISA-TP47, and TPPA maintained positive since seroconversion in both the treatment group and control group. However, one rabbit in the treatment group exhibited sero-negativity in TRUST titer from the 31^st^ day to the 51^st^ day, while the remaining five rabbits remained positive until the 51^st^ day. Interestingly, the LISA-TP15 results in four of six rabbits returned negative after seroconversion, and then became positive once again, we hypothesized that the unstability of LISA-TP15 detection might result from low levels of antibodies against TP15.

Abbreviations: BPG, benzathine penicillin; LISA, luciferase immunosorbent assay; TPPA, *Treponema pallidum* particle agglutination; TRUST, toluidine red unheated serum test.


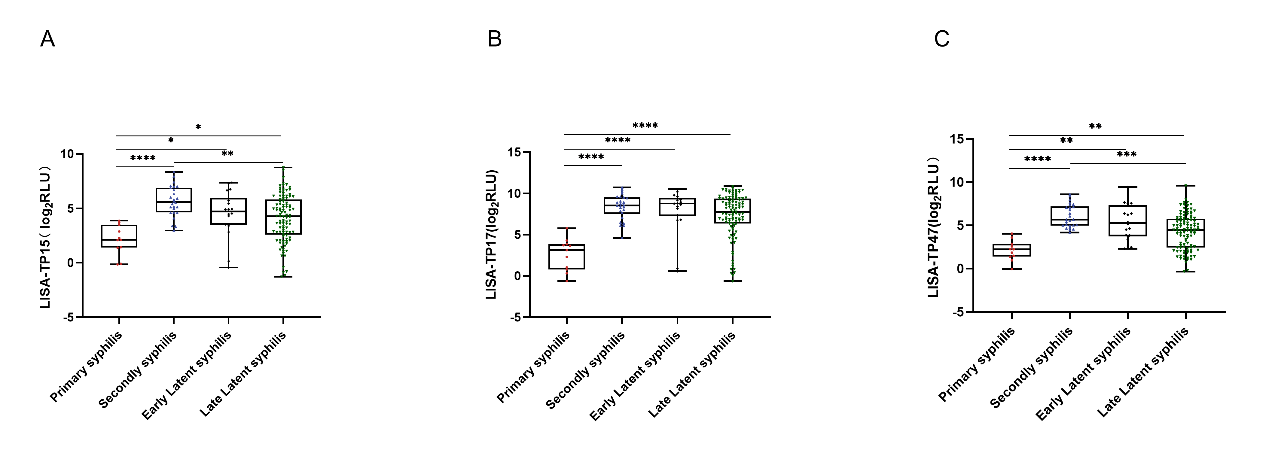


**Supplemental Figure 2 . The level of serum antibodies against TP15, TP17 and TP47 tested by LISA in the primary syphilis, secondary syphilis, early latent syphilis, and late latent syphilis. *****P* < 0.0001;****P* < 0.001;***P* < 0.01;**P* < 0.05.**

Abbreviations: LISA, luciferase immunosorbent assay; RLU, relative light unit.

Reference

1. National Center for STD Control, Chinese Center for Disease Control and Prevention, Venereology Group, Chinese Society of Dermatology, Subcommittee on Venereology, Association. CD. Guidelines for diagnosis and treatment of syphilis, gonorrhea and genital Chlamydia trachomatis infection (2020). Chin J Dermatol **2020**; 53: 168-77.

2. UpToDate. Algorithm for diagnosis of neurosyphilis in a patient without HIV infection. Available at: <https://www.uptodate.com/contents/search?search=Neurosyphilis&sp=0&searchType=PLAIN_TEXT&source=USER_INPUT&searchControl=TOP_PULLDOWN&searchOffset=1&autoComplete=false&language=&max=0&index=&autoCompleteTerm=&rawSentence>=. Accessed 2023/05/23.

3. Workowski KA, Bachmann LH, Chan PA, et al. Sexually Transmitted Infections Treatment Guidelines, 2021. MMWR Recomm Rep **2021**; 70(4): 1-187.

4. Janier M, Unemo M, Dupin N, Tiplica GS, Potocnik M, Patel R. 2020 European guideline on the management of syphilis. J Eur Acad Dermatol Venereol **2021**; 35(3): 574-88.

5. Ke W, Molini BJ, Lukehart SA, Giacani L. Treponema pallidum subsp. pallidum TP0136 protein is heterogeneous among isolates and binds cellular and plasma fibronectin via its NH2-terminal end. PLoS Negl Trop Dis **2015**; 9(3): e0003662.

6. Haynes AM, Godornes C, Ke W, Giacani L. Evaluation of the Protective Ability of the Treponema pallidum subsp. pallidum Tp0126 OmpW Homolog in the Rabbit Model of Syphilis. Infect Immun **2019**; 87(8).

7. Lin LR, Zhu XZ, Liu D, Liu LL, Tong ML, Yang TC. Are nontreponemal tests suitable for monitoring syphilis treatment efficacy? Evidence from rabbit infection models. Clin Microbiol Infect **2020**; 26(2): 240-6.

8. Lukehart SA, Baker-Zander SA, Holmes KK. Efficacy of aztreonam in treatment of experimental syphilis in rabbits. Antimicrobial agents and chemotherapy **1984**; 25(3): 390-1.

9. Baker-Zander SA, Lukehart SA. Efficacy of cefmetazole in the treatment of active syphilis in the rabbit model. Antimicrobial agents and chemotherapy **1989**; 33(9): 1465-9.
